# An Early Warning Marker in Acute Respiratory Failure: The Prognostic Significance of the PaCO_2_–ETCO_2_ Gap During Noninvasive Ventilation

**DOI:** 10.3390/medicina62010197

**Published:** 2026-01-17

**Authors:** Süleyman Kırık, Mehmet Göktuğ Efgan, Ejder Saylav Bora, Uğur Tavşanoğlu, Hüseyin Özkan Öz, Burak Acar, Sedat Yıldızlı

**Affiliations:** 1 Department of Emergency Medicine, Faculty of Medicine, Izmir Katip Çelebi University, İzmir 35100, Türkiye; kiriksuleyman2107@outlook.com (S.K.); saylavbora@hotmail.com (E.S.B.); 2 Department of Emergency Medicine, İstanbul Kanuni Sultan Süleyman Training and Research Hospital, University of Health Sciences, İstanbul 34303, Türkiye; ugurtavsanoglu@gmail.com; 3 Emergency Department, Tosya State Hospital, Kastamonu 37300, Türkiye; ozkanoz_92@hotmail.com; 4 Department of Emergency Medicine, Training and Research Hospital, Adiyaman University, Adiyaman 02030, Türkiye; burakacar7493@gmail.com; 5 Emergency Department, Izmir City Hospital, İzmir 35300, Türkiye; sedatyildizli46@gmail.com

**Keywords:** PaCO_2_–ETCO_2_ gap, respiratory insufficiency, noninvasive ventilation, capnography

## Abstract

*Background and Objectives*: Acute respiratory failure (ARF) has a heterogeneous course in the emergency department (ED), and early prediction of noninvasive mechanical ventilation (NIMV) failure is difficult. The PaCO_2_–ETCO_2_ gap reflects ventilation–perfusion mismatch and increased physiologic dead space; however, the prognostic value of its short-term change during NIMV is unclear. This study evaluated baseline, post-treatment, and delta (post–pre) PaCO_2_–ETCO_2_ gap values for predicting intubation, intensive care unit (ICU) admission, and mortality in ED patients with ARF receiving NIMV. *Materials and Methods*: This prospective observational study enrolled adults (≥18 years) treated with NIMV in a tertiary ED. Exclusion criteria included GCS < 15, intoxication, pneumothorax, trauma, pregnancy, gastrointestinal bleeding, need for immediate intubation/CPR, or incomplete data. ETCO_2_ was recorded within the first 3 min of NIMV and at 30 min; concurrent arterial blood gases provided PaCO_2_. The PaCO_2_–ETCO_2_ gap was calculated at both time points and as delta. Outcomes were intubation, ICU admission, and mortality. ROC analyses determined discriminatory performance and cutoffs using the Youden index. *Results*: Thirty-four patients were included (50% female; mean age 73.26 ± 10.07 years). Intubation occurred in 9 (26.5%), ICU admission in 20 (58.8%), and mortality in 10 (29.4%). The post-treatment gap and delta gap were significantly higher in intubated patients (*p* = 0.007 and *p* = 0.001). For predicting intubation, post-treatment gap > 10.90 mmHg yielded AUC 0.807 (*p* = 0.007; sensitivity 77.8%, specificity 76.0), while delta gap > 2.90 mmHg yielded AUC 0.982 (*p* = 0.001; sensitivity 88.9%, specificity 92.0). Delta gap also predicted ICU admission (cutoff > 0.65 mmHg; AUC 0.746, *p* = 0.016) and mortality (cutoff > 2.90 mmHg; AUC 0.865, *p* = 0.001). *Conclusions*: In ED ARF patients receiving NIMV, an increasing PaCO_2_–ETCO_2_ gap—especially the delta gap—was associated with higher risks of intubation, ICU admission, and mortality, supporting serial CO_2_ gap monitoring as a practical early warning marker of deterioration.

## 1. Introduction

Acute respiratory failure is a serious clinical condition that frequently leads to emergency department visits, can progress rapidly, and carries a high risk of morbidity and mortality [1]. The clinical course in this patient group is quite heterogeneous; while some patients improve rapidly with supportive therapy, others may require invasive mechanical ventilation and intensive care. However, clinical or physiological parameters that can reliably predict the need for intubation, intensive care admission, and mortality in patients presenting with acute respiratory failure have not yet been clearly established. This uncertainty makes it difficult to identify high-risk patients promptly and determine appropriate treatment strategies, especially in emergency department settings.

Non-invasive mechanical ventilation (NIMV) is a proven treatment widely used in emergency departments and intensive care units today for acute respiratory failure. NIMV improves gas exchange by increasing alveolar ventilation, reduces the load on the respiratory muscles, and can reduce the need for endotracheal intubation in appropriate patient groups [2]. It also contributes to the prevention of complications such as infections associated with invasive mechanical ventilation, airway trauma, and the need for sedation. However, when NIMV fails, intubation becomes inevitable, and this situation is associated with a significant increase in mortality risk in patients. Particularly in patients requiring intubation, delayed intubation is associated with worse clinical outcomes and increased mortality [3]. Therefore, it is crucial to predict failure and the need for invasive mechanical ventilation early in patients receiving NIMV.

End-tidal carbon dioxide (ETCO_2_) measurement is a non-invasive, rapid, and easily applied monitoring method used to assess critically ill patients. Capnography is widely used in prehospital and hospital settings to assess ventilation effectiveness, confirm endotracheal tube placement, and monitor respiratory status [4,5]. While ETCO_2_ values in healthy individuals generally correlate closely with arterial partial carbon dioxide pressure (PaCO_2_), this relationship may be disrupted in critically ill patients. In conditions such as ventilation–perfusion mismatch and increased physiological dead space, the difference between PaCO_2_ and ETCO_2_ increases, and this difference is defined as the PaCO_2_–ETCO_2_ gap [6].

Decreased pulmonary perfusion and ventilation–perfusion mismatch cause the PaCO_2_–ETCO_2_ gap to widen. This gap has been shown to increase in many pathological conditions such as pulmonary embolism, obstructive lung diseases, acute respiratory distress syndrome (ARDS), sepsis, traumatic brain injury, and massive bleeding [7]. A significant portion of these clinical presentations comprises patients presenting to the emergency department with shortness of breath. The approach to shortness of breath encompasses a wide range of treatments, from simple oxygen therapy to invasive mechanical ventilation. In this process, it is crucial to avoid unnecessary invasive interventions and to promptly identify patients who require invasive mechanical ventilation.

Recent studies indicate that the PaCO_2_–ETCO_2_ gap may reflect not only the efficacy of ventilation but also tissue perfusion and an increase in physiological dead space. Consequently, it has been indicated that the PaCO_2_–ETCO_2_ gap may correlate with clinical deterioration, the necessity for invasive mechanical ventilation, and mortality in critically ill patients [8,9,10]. Research, particularly involving patients in emergency departments and intensive care units, underscores that an increased PaCO_2_–ETCO_2_ gap may serve as an independent prognostic indicator for unfavourable clinical outcomes [9,10]. All this data substantiates the utilisation of the PaCO_2_–ETCO_2_ difference as a non-invasive, practical, and expeditious prognostic indicator. Nonetheless, the literature contains a limited number of studies examining the prognostic significance of this difference, particularly its alteration during treatment (delta difference), in patients with acute respiratory failure receiving non-invasive mechanical ventilation in the emergency department. This study sought to assess the prognostic significance of the PaCO_2_–ETCO_2_ difference and the delta difference for treatment efficacy, the need for intubation, and mortality in patients with acute respiratory failure presenting to the emergency department who received NIMV.

## 2. Materials and Methods

### 2.1. Study Design

This study was conducted as a prospective, observational clinical trial. The study was conducted at the Emergency Department of Atatürk Training and Research Hospital, İzmir Kâtip Çelebi University. Approval was obtained from the local ethics committee of the relevant university prior to the start of the study.

### 2.2. Study Population

The study population consisted of patients aged 18 years or older who presented to the emergency department with shortness of breath. Patients were included if they required initiation of non-invasive mechanical ventilation due to acute respiratory failure, based on clinical indications consistent with current guidelines, such as increased work of breathing, persistent hypoxemia and/or hypercapnia despite conventional oxygen therapy. Among these patients, those deemed suitable for NIMV and who agreed to participate in the study were included. Patients with a Glasgow Coma Scale (GCS) score < 15, those with a history of alcohol or drug use, those with pneumothorax or suspected pneumothorax, those with a history of trauma, those who were pregnant, those diagnosed with gastrointestinal bleeding, those requiring emergency intubation or cardiopulmonary resuscitation upon arrival at the emergency department, and those with incomplete clinical or laboratory data were excluded from the study. Patients were enrolled using a non-probability convenience sampling method, including consecutive eligible patients presenting to the emergency department during the study period.

### 2.3. Study Protocol and Data Collection

Standard monitoring was provided after the initiation of mechanical ventilation in patients who presented to the emergency department with shortness of breath and who underwent non-invasive mechanical ventilation in accordance with guideline recommendations. Monitoring included continuous measurement of systolic and diastolic arterial blood pressure, pulse rate, respiratory rate, and ETCO_2_ values. Patients’ non-invasive mechanical ventilation treatments were continued in 30 min periods; at the end of each 30 min period, non-invasive ventilation was paused briefly, and the response to treatment was routinely evaluated with arterial blood gas analysis.

For the patients participating in the study, the ETCO_2_ values recorded within the initial 3 min of non-invasive mechanical ventilation and at the 30 min mark, as well as the partial carbon dioxide pressure (PaCO_2_) values from arterial blood gas samples obtained concurrently at these intervals, were documented on the data recording form. Noninvasive ventilation was administered using standard bilevel NIV devices with intentional leak ports, in accordance with manufacturer recommendations. Quantitative inspiratory and expiratory tidal volume measurements or numerical leak values were not routinely available from the ventilators used in this study. To minimise the impact of mask leak on ETCO_2_ measurements, NIV was applied by experienced clinicians with careful mask fitting and continuous bedside monitoring. ETCO_2_ waveforms were continuously observed, and only measurements with stable and well-defined capnogram waveforms were recorded. Patients with clinically evident excessive mask leak, poor mask tolerance, or unstable capnography tracings were excluded from analysis. Arterial blood gas sampling and ETCO_2_ measurements were obtained concurrently under identical ventilatory conditions. To prevent treatment delays, non-invasive mechanical ventilation was commenced prior to arterial blood gas and end-tidal measurements, which were obtained within the first 3 min. Furthermore, essential parameters, hemogram and biochemical laboratory results, and arterial blood gas metrics, including oxygen saturation (SaO_2_), partial oxygen pressure (PaO_2_), partial carbon dioxide pressure (PaCO_2_), lactate concentrations, and ETCO_2_ measurements, were documented, alongside the necessity for intubation, intensive care unit admission status, and mortality status.

### 2.4. Outcomes

The decision to perform endotracheal intubation and initiate invasive mechanical ventilation was based on clinical criteria indicating noninvasive ventilation failure, including worsening respiratory distress, progressive hypoxemia or hypercapnia, deterioration in mental status, hemodynamic instability, or inability to protect the airway. These decisions were made by the attending emergency physician in accordance with standard clinical practice.

### 2.5. Statistical Analysis

Statistical analysis of the data was conducted utilising IBM SPSS Statistics 25.0 (IBM Corp., Armonk, NY, USA). The distribution characteristics of continuous variables were assessed through visual methods (histograms and Q–Q plots) and analytical techniques. Continuous variables exhibiting a normal distribution were reported as mean ± standard deviation, whereas continuous variables not exhibiting a normal distribution were reported as median (minimum–maximum). Categorical variables were represented as numerical values and percentages (%). Patients were categorised into two groups based on clinical outcomes: intubation (yes/no), intensive care unit admission (yes/no), and mortality (yes/no). The independent-samples Student *t*-test or the Mann–Whitney U test was used to compare continuous variables between the two groups, depending on their distributions. The Pearson chi-square test was employed to compare categorical variables. The PaCO_2_–ETCO_2_ difference was determined as the absolute disparity between the PaCO_2_ value obtained from arterial blood gas analysis and the concurrently measured end-tidal CO_2_ value. Values for pre-treatment, post-treatment, and delta (post-treatment minus pre-treatment) were analysed for this difference. Receiver operating characteristic (ROC) curve analyses were conducted to assess the diagnostic efficacy of the PaCO_2_–ETCO_2_ difference in forecasting the necessity for intubation, admission to the intensive care unit, and mortality. The area under the curve (AUC) and 95% confidence intervals were computed in the ROC analyses. The ideal cut-off points were established utilising the Youden index; sensitivity and specificity metrics were computed for each cut-off point. In all statistical analyses, a two-tailed *p*-value of less than 0.05 was deemed statistically significant.

## 3. Results

The study included 34 patients. Seventeen patients (50.0%) were female. The average age was 73.26 ± 10.07 years. The initial end-tidal CO_2_ measurement was 34.76 ± 12.51 mmHg, the PaCO_2_ measurement was 44.29 ± 16.49 mmHg, and the PaCO_2_–ETCO_2_ differential was 9.35 ± 4.30 mmHg. At the 30th minute of the second measurement, the end-tidal CO_2_ value was 36.64 ± 11.41 mmHg, the PaCO_2_ value was 40.34 ± 13.12 mmHg, and the PaCO_2_–ETCO_2_ difference was 9.40 ± 7.21 mmHg. The computed mean delta difference was 0.12 ± 8.12 mmHg.

Seven patients (20.6%) were discharged from the emergency department, whereas 27 patients (79.4%) were not. Nine patients (26.5%) necessitated intubation during follow-up. Twenty patients (58.8%) were admitted to the intensive care unit, whereas 14 patients (41.2%) did not necessitate such admission. Mortality occurred in 10 patients (29.4%). Descriptive results are displayed in Table 1.

Patients were classified into two groups based on their requirement for intubation, and a comparative analysis was performed between each group (Table 2). No statistically significant differences were detected between the groups for age, blood pressure (systolic and diastolic), pulse rate, respiratory rate, end-tidal CO_2_, PaCO_2_, oxygen saturation, and PaO_2_ in both initial and subsequent measurements (*p* > 0.05).

The body temperature upon admission was elevated in the group that required intubation, and this difference was statistically significant (*p* = 0.013). Lactate levels were generally elevated in the group that required intubation at the initial measurement; however, this disparity did not achieve statistical significance (*p* = 0.069).

In the second measurement, pH values were lower in the intubated group (*p* = 0.006). Lactate levels were significantly elevated in the group that required intubation at the second measurement (*p* = 0.037). The HCO_3_^−^ levels were markedly reduced in the intubated group during the second measurement (*p* = 0.014).

The PaCO_2_–ETCO_2_ difference was markedly elevated in the intubated group during the second measurement (*p* = 0.007). Moreover, the PaCO_2_–ETCO_2_ delta difference exhibited a significant increase in the intubated group, whereas a decrease was noted in the non-intubated group, with this disparity being statistically significant (*p* = 0.001).

ROC analysis was performed on parameters related to the PaCO_2_–ETCO_2_ difference to forecast the requirement for intubation (Table 3). The discriminatory ability of the pre-treatment PaCO_2_–ETCO_2_ difference was negligible, with an AUC of 0.458 (*p* = 0.711). The examination of the PaCO_2_–ETCO_2_ difference following treatment demonstrated a notable improvement in discriminatory capability, indicated by an AUC of 0.807 (*p* = 0.007). The sensitivity for this parameter was 77.8%, and the specificity was 76.0% at a threshold exceeding 10.90 mmHg.

**Figure 1:**
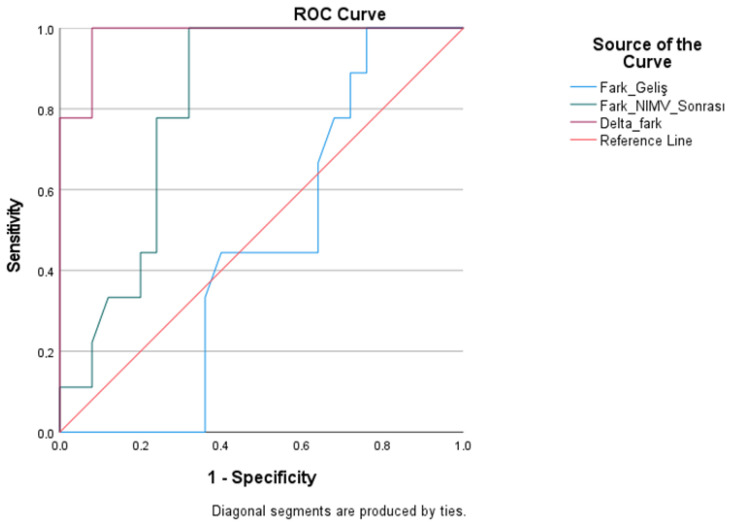
ROC Curve of the Diagnostic Performance of the PaCO_2_–ETCO_2_ Difference in Predicting the Need for Intubation.

The delta PaCO_2_–ETCO_2_ difference demonstrated the highest diagnostic effectiveness in predicting the need for intubation. The area under the curve (AUC) for this variable was 0.982 and statistically significant (*p* = 0.001). At a threshold exceeding 2.90 mmHg, sensitivity was 88.9% and specificity was 92.0%. Figure 1 illustrates the ROC curves.

Patients were categorised into two groups according to their need for intensive care admission and subsequently compared (Table 4). No statistically significant differences were observed between the groups regarding age, systolic and diastolic blood pressure, pulse rate, respiratory rate, and end-tidal CO_2_, PaCO_2_, and HCO_3_^−^ values in the initial and subsequent measurements (all *p* > 0.05).

The initial body temperature was markedly elevated in the cohort, necessitating intensive care admission (*p* = 0.010). The second measurement of oxygen saturation was markedly reduced in the group necessitating intensive care admission (*p* = 0.020). The second PaO_2_ measurement was significantly lower in the group necessitating intensive care admission (*p* = 0.025).

The delta value of the PaCO_2_–ETCO_2_ difference was markedly elevated in the cohort necessitating intensive care admission (*p* = 0.016). No statistically significant differences were detected between the groups concerning other PaCO_2_–ETCO_2_ differential measurements.

ROC analysis was conducted for parameters associated with the PaCO_2_–ETCO_2_ difference to predict the necessity for intensive care admission (Table 5). The discriminatory capability of the pre-treatment PaCO_2_–ETCO_2_ difference was minimal, with an AUC of 0.462 (*p* = 0.713). The analysis of the PaCO_2_–ETCO_2_ difference post-treatment revealed a moderate discriminatory power, indicated by an AUC value of 0.680; nonetheless, this finding did not achieve statistical significance (*p* = 0.077).

**Figure 2:**
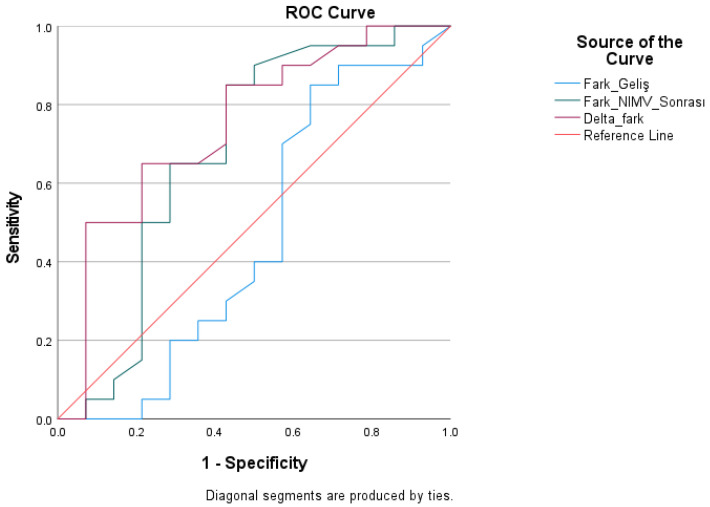
ROC Curve of the Diagnostic Performance of the PaCO_2_–ETCO_2_ Difference in Predicting the Need for Intensive Care Unit Admission.

The delta PaCO_2_–ETCO_2_ difference demonstrated statistically significant efficacy in predicting the necessity for intensive care admission. The AUC for this variable was 0.746 and statistically significant (*p* = 0.016). At a threshold of >0.65 mmHg, sensitivity was 60.0% and specificity was 78.6%. Figure 2 displays the ROC curves.

Patients were categorised into two groups according to mortality status, and a comparison was conducted between the groups (Table 6). No statistically significant differences were observed between the groups regarding age, systolic and diastolic arterial blood pressure, respiratory rate, end-tidal CO_2_, PaCO_2_, oxygen saturation, and PaO_2_ in the initial and subsequent measurements (all *p* > 0.05).

The admission pulse rate was markedly elevated in the cohort that experienced mortality (*p* = 0.033). The body temperature in the same group was significantly elevated (*p* = 0.040).

The initial HCO_3_^−^ levels were markedly reduced in the mortality group (*p* = 0.038). HCO_3_^−^ levels were markedly diminished in the mortality group during the second measurement (*p* = 0.033).

The delta value of the PaCO_2_–ETCO_2_ difference was markedly elevated in the group that experienced mortality (*p* = 0.001). No statistically significant differences were noted between the groups regarding other PaCO_2_–ETCO_2_ differential measurements.

ROC analysis was conducted for parameters associated with the PaCO_2_–ETCO_2_ difference in forecasting mortality (Table 7). The PaCO_2_–ETCO_2_ difference before treatment exhibited minimal discriminatory capability for mortality, with an AUC of 0.383 (*p* = 0.290). The analysis of the PaCO_2_–ETCO_2_ difference post-treatment demonstrated limited discriminatory capability, with an AUC of 0.713; however, this difference did not achieve statistical significance (*p* = 0.054).

**Figure 3:**
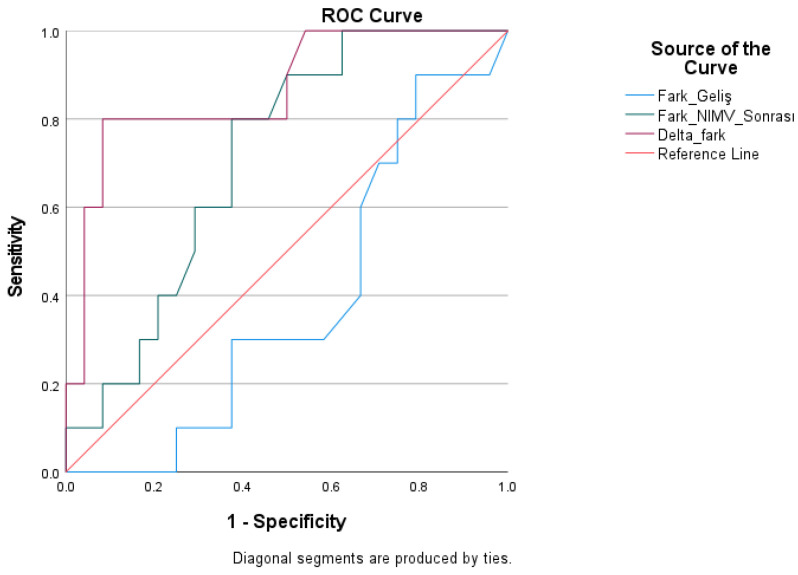
ROC Curve for the Diagnostic Performance of PaCO_2_–ETCO_2_ Difference in Predicting Mortality.

The delta PaCO_2_–ETCO_2_ discrepancy demonstrated significant diagnostic efficacy in forecasting mortality. The area under the curve (AUC) for this variable was 0.865 and was statistically significant (*p* = 0.001). At a threshold of >2.90 mmHg, sensitivity was 80.0% and specificity was 91.7%. Figure 3 displays the ROC curves.

## 4. Discussion

This study investigated the prognostic significance of the arterial–end-tidal carbon dioxide difference (PaCO_2_–ETCO_2_), or CO_2_ deficit, in patients receiving noninvasive mechanical ventilation (NIMV) for acute respiratory failure in the emergency department. The findings demonstrate that an increased delta PaCO_2_–ETCO_2_ difference after treatment is significantly associated with a higher risk of intubation, intensive care unit (ICU) admission, and mortality. These results suggest that the CO_2_ difference may serve as a dynamic and noninvasive physiological marker that reflects both ventilation efficiency and perfusion abnormalities, providing early insight into treatment response.

Physiologically, the widening of the PaCO_2_–ETCO_2_ difference is known to indicate increased alveolar dead space, impaired gas exchange, and reduced pulmonary blood flow. Early studies by Nunn & Hill [6] and Askrog [9] established that the CO_2_ gap reflects diminished pulmonary perfusion, findings that remain relevant today. In the present study, patients who developed mortality exhibited both a greater CO_2_ difference and lower HCO_3_^−^ levels, supporting the association between widening CO_2_ gaps, tissue hypoxia, and metabolic acidosis. This relationship highlights the potential of the CO_2_ difference as an integrative indicator of both ventilatory and circulatory dysfunction.

İşat et al. demonstrated a strong correlation between arterial PaCO_2_ and ETCO_2_ in patients with acute COPD exacerbation, suggesting that high ETCO_2_ values may indicate the need to initiate noninvasive mechanical ventilation early, without waiting for blood gas results [10]. Likewise, Bhattacharyya found that improvements in respiratory rate, heart rate, pH, and PaCO_2_ after 1 h of NIMV predict successful outcomes in acute type 2 respiratory failure [11]. These studies reinforce the importance of continuous CO_2_ monitoring and early assessment of physiological responses to guide timely and effective management during NIMV.

The current results align closely with prior research. Shetty et al. [12] and Thacker et al. [8] reported that a CO_2_ gap exceeding 5–10 mmHg was independently associated with mortality in patients presenting with sepsis or undergoing intensive care, respectively. Similarly, Abdalrazik and Elghonemi [13] found that a large CO_2_ difference correlated with disease severity and mortality in patients with acute respiratory distress syndrome (ARDS). Our finding that a delta difference exceeding 2.9 mmHg predicted adverse outcomes with high sensitivity and specificity supports the same clinical principle, though at a lower threshold. This may reflect the inclusion of patients at earlier stages of respiratory failure or differences in NIMV settings. Although a delta PaCO_2_–ETCO_2_ difference greater than 2.9 mmHg demonstrated strong discriminatory performance in this cohort, this value should not be interpreted as a definitive threshold for intubation. Given the small sample size and non-probability sampling design, the delta difference should be regarded as an early physiological warning marker rather than a stand-alone trigger for invasive mechanical ventilation. Reliance on small numerical changes in isolation may lead to premature intubation and unnecessary exposure to invasive ventilation-related complications. Instead, an increasing PaCO_2_–ETCO_2_ delta should prompt closer clinical monitoring, reassessment of noninvasive ventilation efficacy, and integration with established clinical indicators of respiratory failure. Larger, multicenter studies are required to validate optimal cutoff values and to determine how this parameter can be safely incorporated into clinical decision-making algorithms. Furthermore, the correlation between increasing CO_2_ difference and treatment failure in our study parallels the results of Defilippis et al. [14], who reported that a decreasing CO_2_ difference during NIMV signalled treatment success. Taken together, these studies suggest that serial monitoring of the CO_2_ difference provides more meaningful prognostic information than a single measurement, as dynamic changes reflect evolving ventilation–perfusion status.

The pathophysiological mechanisms underlying this relationship are well established. As ventilation–perfusion mismatch progresses, the correlation between ETCO_2_ and PaCO_2_ deteriorates due to increased alveolar dead space, as demonstrated by Razi et al. [7]. In this study, patients requiring intubation exhibited a marked widening of the CO_2_ difference following treatment, indicating worsening mismatch and ineffective NIMV response. These findings underscore that the CO_2_ difference reflects not only ventilation adequacy but also perfusion at the microcirculatory level, as supported by Davis et al. [15], who found that high intraoperative CO_2_ differences were associated with postoperative complications and mortality. Moreover, Hong et al. [16] demonstrated that the arterial–end-tidal CO_2_ gradient could serve as a reliable index of disease severity in critically ill patients, further confirming its prognostic value across diverse clinical settings. Similarly, Masoumi et al. [17] observed a strong correlation between PaCO_2_ and ETCO_2_ in patients with respiratory distress, while mortality tended to increase with a larger CO_2_ gap, reinforcing the current study’s findings.

Recent trauma research adds further context. Sardesai et al. reported that the PaCO_2_–ETCO_2_ gradient in adult trauma patients with TBI was greater than previously described and associated with increased mortality early in care [18]. Likewise, Upchurch et al. found that an elevated PaCO_2_–ETCO_2_ gap is common post-intubation in the emergency department, though it is not significantly associated with outcomes [19]. These results emphasise that the clinical implications of CO_2_ gradients vary depending on patient population, timing, and physiological context.

Nevertheless, some studies have questioned the reliability of the CO_2_ difference as a prognostic marker. Kodali [4] emphasised that capnograph readings during noninvasive ventilation may be affected by technical factors, including mask leakage, patient cooperation, and sampling method. Supporting this, Sakuraya et al. [20] demonstrated that mainstream capnography correlates more strongly with PaCO_2_ than side-stream measurements, suggesting that methodological factors substantially influence accuracy. This limitation was also acknowledged in our study, as measurement variability could have affected precision. In addition, the relatively small sample size and single-centre design may limit the generalizability of our results.

Another important consideration is the potential influence of underlying disease severity and aetiology. For example, Carrillo et al. [3] found that noninvasive ventilation was effective in selected patients with severe community-acquired pneumonia and acute respiratory failure, if treatment response was closely monitored. Their findings align with ours, indicating that dynamic physiological markers, such as the CO_2_ difference, can help clinicians identify NIMV failure early and avoid delayed intubation in high-risk patients.

In addition to the clinical correlations previously discussed, several studies provide further support for the utility and complexity of PaCO_2_ and ETCO_2_ measurements in both research and clinical settings [5]. Rentola et al. demonstrated that while noninvasive estimation of arterial CO_2_ using end-tidal values is feasible, its accuracy varies significantly with physiological and hemodynamic states, underscoring the need for careful interpretation of the PaCO_2_–ETCO_2_ gradient in unstable patients [20]. This aligns with our findings that variations in CO_2_ gradients reflect underlying ventilation–perfusion mismatch and may predict clinical deterioration.

Furthermore, monitoring strategies during noninvasive respiratory support emphasise that serial, multimodal physiological monitoring beyond simple CO_2_ measurements can improve the early identification of patients at risk of progression to respiratory failure [21]. Pelosi et al. reviewed physiological monitoring in acute respiratory distress syndrome and highlighted the importance of integrated respiratory parameters to optimise noninvasive ventilation and avoid prolonged ineffective support, suggesting a broader interpretive framework that includes trends in CO_2_ values rather than single measurements [22]. This supports our suggestion that dynamic changes in PaCO_2_–ETCO_2_ differences are more informative than static cutoffs.

Despite these limitations, our findings reinforce the potential clinical value of the CO_2_ difference as an early, accessible, and noninvasive predictor of NIMV outcomes. Continuous or serial measurement of this parameter could help identify patients at high risk of treatment failure, allowing timely escalation of care before overt clinical deterioration. Future studies with larger, multicentre cohorts and standardised capnography techniques are needed to validate optimal cutoff values and integrate this marker into predictive scoring systems for respiratory failure.

Second, quantitative ventilator-derived measurements of inspiratory–expiratory tidal volume differences and numerical leak values were not available, which may limit direct assessment of mask leak during noninvasive ventilation. However, strict clinical monitoring, waveform-based validation of capnography, and the use of dynamic PaCO_2_–ETCO_2_ changes rather than isolated ETCO_2_ values likely mitigated this limitation.

## 5. Conclusions

In conclusion, the PaCO_2_–ETCO_2_ difference represents a promising physiological indicator that bridges respiratory and circulatory assessment in patients undergoing NIMV. A rising CO_2_ difference during treatment may signal early treatment failure and increased mortality risk, while a decreasing difference may indicate therapeutic success. Incorporating dynamic CO_2_ monitoring into clinical practice could enhance early decision-making, optimise patient management, and potentially improve outcomes in acute respiratory failure. Future multicentre prospective studies are warranted to determine standardised PaCO_2_–ETCO_2_ thresholds that can be incorporated into predictive models to detect early NIMV failure.

## Figures and Tables

**Table 1:** Descriptive Characteristics (n = 34).

	Statistics
**Gender**	
Woman	17 (50.0)
Male	17 (50.0)
Age *	73.26 ± 10.07
Systolic Blood Pressure *	132.20 ± 28.12
Diastolic TA *	74.38 ± 15.11
Pulse *	106.88 ± 25.70
Respiratory Rate *	30.08 ± 5.90
Temperature *	36.46 ± 0.29
End Tidal Measurement 1 *	34.76 ± 12.51
pH 1. Measurement *	7.35 ± 0.10
PaCO_2_ Measurement 1 *	44.29 ± 16.49
Saturation 02 1st Measurement *	87.00 ± 14.43
PaO_2_ Measurement 1 *	80.47 ± 25.78
Lactate Measurement 1 *	3.09 ± 2.33
HCO_3_ 1st Measurement *	22.91 ± 4.86
Difference 1. Measurement *	9.52 ± 9.43
End Tidal Measurement 2 *	33.64 ± 11.41
pH 2. Measurement *	7.36 ± 0.12
PaCO_2_ Measurement 2 *	43.04 ± 13.12
Saturation 02 2nd Measurement *	92.65 ± 10.23
PaO_2_ Measurement 2 *	94.86 ± 37.33
Lactate Measurement 2 *	2.87 ± 2.37
HCO_3_ 2nd Measurement *	23.07 ± 5.24
Difference 2. Measurement *	9.40 ± 7.21
Delta Difference *	0.12 ± 8.12
**Emergency room discharge,** ***n*** **(%)**	
**Yes**	7 (20.6)
**No**	27 (79.4)
**Type of respiratory failure**	
**Type 1**	24 (70.6)
**Type 2**	10 (29.4)
**Intubation**	
**Yes**	9 (26.5)
**No**	25 (73.5)
**Intensive Care Unit Admission**	
**Yes**	20 (58.8)
**No**	14 (41.2)
**Mortality**	
**None**	24 (70.6)
**Exitus**	10 (29.4)

Values: Number of patients, %: Column percentage, *: mean ± standard deviation.

**Table 2:** Comparison of Clinical, Vital, and Laboratory Parameters in Patients Who Developed and Did Not Develop a Need for Intubation.

	Intubation		
	No	There	*p*
**Age**	73.00 ± 10.87 76.00 (51.00–92.00)	74.00 ± 7.96 74.00 (62.00–86.00)	0.860 ^+^
**Systolic Blood Pressure**	136.48 ± 29.94 138.00 (84.00–192.00)	120.33 ± 18.94 113.00 (95.00–155.00)	0.171 *
**Diastolic Blood Pressure**	75.72 ± 16.30 76.00 (48.00–112.00)	70.66 ± 11.10 70.00 (57.00–90.00)	0.423 *
**Pulse**	105.56 ± 25.73 110.00 (58.00–158.00)	110.55 ± 26.78 108.00 (65.00–160.00)	0.696 *
**Respiratory Rate**	30.44 ± 6.10 30.00 (20.00–44.00)	29.11 ± 5.51 28.00 (21.00–38.00)	0.595 *
**Temperature**	36.41 ± 0.30 36.40 (36.00–37.20)	36.61 ± 0.22 36.50 (36.40–37.10)	0.013 ^+^
**End Tidal Measurement 1**	35.44 ± 10.81 36.00 (20.00–53.00)	32.88 ± 17.03 30.00 (11.00–58.00)	0.639 *
**pH 1. Measurement**	7.35 ± 0.10 7.38 (7.08–7.53)	7.32 ± 0.11 7.30 (7.22–7.50)	0.329 *
**PaCO_2_ Measurement 1**	46.06 ± 16.53 41.00 (24.20–85.00)	39.38 ± 16.27 38.50 (20.20–63.00)	0.274 ^+^
**Saturation 02 1st Measurement**	86.99 ± 15.51 93.00 (38.20–98.60)	87.04 ± 11.71 93.30 (70.30–98.00)	0.922 ^+^
**PaO_2_ Measurement 1**	81.67 ± 24.73 75.20 (48.50–140.00)	77.12 ± 29.83 66.70 (47.00–130.00)	0.401 *
**Lactate Measurement 1**	2.76 ± 2.46 1.70 (0.50–8.90)	4.01 ± 1.72 4.70 (0.80–5.50)	0.069 ^+^
**HCO_3_ 1st Measurement**	24.15 ± 4.51 24.20 (15.00–34.80)	19.47 ± 4.28 18.90 (15.40–29.00)	0.012 *
**Difference 1. Measurement**	10.62 ± 10.75 7.10 (0.20–46.00)	6.50 ± 2.42 6.60 (2.20–9.20)	0.711 ^+^
**End Tidal Measurement 2**	35.16 ± 11.22 32.00 (18.00–60.00)	29.44 ± 11.52 27.00 (11.00–51.00)	0.218 *
**pH 2. Measurement**	7.39 ± 0.11 7.40 (7.10–7.72)	7.21 ± 0.08 7.25 (7.21–7.48)	0.006 ^+^
**PaCO_2_ Measurement 2**	42.73 ± 13.22 41.50 (26.70–72.50)	43.91 ± 13.56 39.80 (22.80–67.50)	0.725 ^+^
**Saturation 02 2nd Measurement**	94.48 ± 9.72 96.00 (60.40–120.00)	87.56 ± 10.43 91.00 (71.00–99.00)	0.075 ^+^
**PaO_2_ Measurement 2**	100.74 ± 40.35 91.50 (48.00–223.00)	78.54 ± 21.40 72.50 (56.90–120.00)	0.105 ^+^
**Lactate Measurement 2**	2.30 ± 2.13 1.40 (0.40–8.50)	4.45 ± 2.42 5.80 (1.00–6.80)	0.037 ^+^
**HCO_3_ 2nd Measurement**	24.48 ± 4.67 25.00 (14.70–34.00)	19.15 ± 4.93 18.10 (14.20–29.40)	0.014 *
**Difference 2. Measurement**	7.57 ± 6.89 5.00 (0.00–25.00)	14.46 ± 5.70 12.00 (9.00–26.00)	0.007 ^+^
**Delta Difference**	−3.04 ± 6.93 −1.00 ((−22.50)–4.00)	7.96 ± 5.24 6.90 (2.80–17.00)	0.001 ^+^

Continuous variables are presented as mean ± standard deviation and median (minimum–maximum). Normality of data distribution was assessed using visual and analytical methods. For group comparisons, variables with normal distribution were analysed using Student’s *t*-test, and the corresponding *p* values are indicated with ‘’*’’. Variables with non-normal distribution were analysed using the Mann–Whitney U test, and the corresponding *p* values are indicated with ‘’^+^’’. All tests were two-tailed, and a *p* value < 0.05 was considered statistically significant.

**Table 3:** ROC Analysis of the Diagnostic Performance of Parameters Related to the PaCO_2_–ETCO_2_ Difference in Predicting the Need for Intubation.

Test Result Variables	*Cutoff*	AUC	Std. Error	Youden Index J	*p*	Lower Bound	Upper Bound	*Sensitivity*	*Specificity*
**Pre-treatment difference**	>6.30	0.458	0.098	−0.09	0.711	0.265	0.650	55.60	36.00
**Post-Treatment Difference**	>10.90	0.807	0.073	0.53	0.007	0.663	0.950	77.80	76.00
**Delta Difference**	>2.90	0.982	0.018	0.80	0.001	0.947	0.1000	88.90	92.00

AUC: Area Under the Curve. ROC analysis was performed to predict the need for intubation based on pre-treatment, post-treatment, and delta values of the PaCO_2_–ETCO_2_ difference. The optimal cutoff point was determined using the Youden index. Sensitivity and specificity values are presented as percentages (%). Statistical significance was accepted at *p* < 0.05.

**Table 4:** Comparison of Clinical, Vital, and Laboratory Parameters of Patients Requiring and Not Requiring Intensive Care Unit Admission.

	Intensive Care Unit Admission		
	No	Yes	*p*
**Age**	73.78 ± 10.15 75.50 (51.00–92.00)	72.90 ± 10.26 75.00 (51.00–86.00)	0.944 *
**Systolic blood pressure**	140.42 ± 27.95 139.00 (100.00–192.00)	126.45 ± 27.47 117.00 (84.00–188.00)	0.151 *
**Diastolic TA**	79.50 ± 17.10 79.00 (48.00–112.00)	70.80 ± 12.79 67.00 (53.00–98.00)	0.096 *
**Pulse**	99.14 ± 25.56 9.00 (58.00–140.00)	112.30 ± 25.00 114.00 (65.00–160.00)	0.208 *
**Respiratory Rate**	30.57 ± 6.68 30.00 (20.00–44.00)	29.75 ± 5.43 29.50 (20.00–41.00)	0.832 *
**Temperature**	36.35 ± 0.28 36.40 (36.00–37.20)	36.54 ± 0.28 36.50 (36.00–37.10)	0.010 ^+^
**End Tidal Measurement 1**	36.00 ± 9.93 39.00 (22.00–51.00)	33.90 ± 14.23 30.00 (11.00–58.00)	0.661 *
**pH 1. Measurement**	7.35 ± 0.09 7.36 (7.08–7.48)	7.34 ± 0.11 7.35 (7.19–7.53)	0.806 *
**PaCO_2_ Measurement 1**	47.65 ± 17.33 40.60 (31.00–85.00)	41.94 ± 15.89 39.25 (20.20–71.70)	0.302 ^+^
**Saturation 02 1st Measurement**	88.80 ± 10.72 93.40 (62.00–98.00)	85.75 ± 16.69 92.85 (38.20–98.60)	0.972 ^+^
**PaO_2_ Measurement 1**	73.70 ± 24.26 68.55 (47.00–140.00)	85.20 ± 26.35 79.80 (49.10–130.00)	0.208 ^+^
**Lactate Measurement 1**	2.42 ± 2.11 1.60 (0.50–8.20)	3.56 ± 2.42 3.45 (0.60–8.90)	0.183 ^+^
**HCO_3_ 1st Measurement**	23.84 ± 3.76 24.10 (18.20–30.00)	22.27 ± 5.50 20.70 (15.00–34.80)	0.294 *
**Difference 1. Measurement**	11.65 ± 13.11 7.70 (0.20–46.00)	8.04 ± 5.58 7.00 (0.20–21.00)	0.713 ^+^
**End Tidal Measurement 2**	36.28 ± 11.17 34.50 (21.00–60.00)	31.80 ± 11.49 28.00 (11.00–54.00)	0.213 *
**pH 2. Measurement**	7.36 ± 0.09 7.37 (7.10–7.48)	7.36 ± 0.13 7.34 (7.18–7.72)	0.687 *
**PaCO_2_ Measurement 2**	44.06 ± 12.54 42.20 (28.20–72.50)	42.33 ± 13.78 38.90 (22.80–69.70)	0.575 *
**Saturation 02 2nd Measurement**	95.91 ± 10.66 98.20 (71.00–120.00)	90.36 ± 9.53 93.65 (60.40–99.00)	0.020 ^+^
**PaO_2_ Measurement 2**	112.90 ± 47.21 102.00 (52.10–223.00)	82.24 ± 22.10 81.25 (48.00–139.00)	0.025 *
**Lactate Measurement 2**	2.17 ± 1.94 1.30 (0.40–6.60)	3.36 ± 2.57 2.25 (0.60–8.50)	0.278 ^+^
**HCO_3_ 2nd Measurement**	24.02 ± 4.30 24.85 (17.50–29.50)	22.41 ± 5.82 22.10 (14.20–34.00)	0.344 *
**Difference 2. Measurement**	7.77 ± 8.43 3.50 (0.00–26.00)	10.53 ± 6.19 10.70 (0.70–25.00)	0.077 ^+^
**Delta Difference**	−3.87 ± 10.26 −1.60 ((−22.50)–17.00)	2.49 ± 4.89 2.25 ((−6.30)–16.00)	0.016 ^+^

Continuous variables are presented as mean ± standard deviation and median (minimum–maximum). Normality of data distribution was assessed using visual and analytical methods. For group comparisons, variables with normal distribution were analyzed using the Student’s *t*-test, and the corresponding p values are indicated with ‘’*’’. Variables with non-normal distribution were analyzed using the Mann–Whitney U test, and the corresponding *p* values are indicated with ‘’^+^’’. All tests were two-tailed, and a *p* value < 0.05 was considered statistically significant.

**Table 5:** ROC Analysis of the Diagnostic Performance of Parameters Related to the PaCO_2_–ETCO_2_ Difference in Predicting the Need for Intensive Care Unit Admission.

Test Result Variables	*Cutoff*	AUC	Std. Error	Youden Index J	*p*	Asymptotic 95% Confidence Interval	Asymptotic 95% Confidence Interval	*Sensitivity*	*Specificity*
**Pre-treatment difference**	>7.05	0.462	0.111	−0.13	0.713	0.245	0.680	45.00	42.90
**Post-Treatment Difference**	>8.75	0.680	0.104	0.36	0.077	0.476	0.884	65.00	71.40
**Delta Difference**	>0.65	0.746	0.089	0.38	0.016	0.572	0.921	60.00	78.60

AUC: Area Under the Curve. ROC analysis was performed to predict the need for intensive care unit admission using pre-treatment, post-treatment, and delta values of the PaCO_2_–ETCO_2_ difference. The optimal cutoff point was determined using the Youden index. Sensitivity and specificity values are presented as percentages (%). The statistical significance level was set at *p* < 0.05.

**Table 6:** Comparison of Clinical, Vital, and Laboratory Parameters of Patients Who Developed and Did Not Develop Mortality.

	Mortality		
	No	Yes	*p*
**Age**	72.97 ± 10.84 75.50 (51.00–92.00)	74.40 ± 8.35 73.50 (61.00–86.00)	0.970 ^+^
**Systolic blood pressure**	136.12 ± 30.55 135.50 (84.00–192.00)	122.80 ± 19.37 117.00 (95.00–155.00)	0.316 *
**Diastolic Blood Pressure**	74.58 ± 16.40 72.00 (48.00–112.00)	73.90 ± 12.21 76.00 (57.00–90.00)	0.895 *
**Pulse**	100.70 ± 21.55 101.00 (58.00–140.00)	121.70 ± 29.82 122.50 (65.00–160.00)	0.033 *
**Respiratory Rate**	29.83 ± 6.39 29.00 (20.00–44.00)	30.70 ± 4.73 30.00 (21.00–38.00)	0.446 *
**Temperature**	36.41 ± 0.30 36.40 (36.00–37.20)	36.58 ± 0.25 36.50 (36.20–37.10)	0.040 ^+^
**End Tidal Measurement 1**	34.70 ± 11.87 37.50 (11.00–53.00)	34.90 ± 14.63 32.00 (14.00–58.00)	0.925 *
**pH 1. Measurement**	7.35 ± 0.10 7.37 (7.08–7.53)	7.33 ± 0.10 7.33 (7.22–7.50)	0.416 *
**PaCO_2_ Measurement 1**	45.49 ± 16.78 40.60 (20.20–85.00)	41.41 ± 16.27 39.25 (20.60–67.60)	0.472 *
**Saturation 02 1st Measurement**	85.69 ± 15.98 92.70 (38.20–98.00)	90.16 ± 9.73 93.65 (72.00–98.60)	0.438 ^+^
**PaO_2_ Measurement 1**	78.60 ± 24.81 73.40 (48.50–140.00)	84.94 ± 28.86 79.85 (47.00–130.00)	0.650 ^+^
**Lactate Measurement 1**	2.94 ± 2.47 2.00 (0.50–8.90)	3.46 ± 2.03 3.85 (0.60–5.50)	0.438 ^+^
**HCO_3_ 1st Measurement**	23.92 ± 4.21 24.60 (15.00–30.90)	20.50 ± 5.65 20.10 (15.40–34.80)	0.038 ^+^
**Difference 1. Measurement**	10.78 ± 10.68 7.65 (00.20–46.00)	6.51 ± 4.46 6.30 (0.20–16.60)	0.290 ^+^
**End Tidal Measurement 2**	35.04 ± 11.22 31.00 (18.00–60.00)	30.30 ± 11.75 27.50 (11.00–54.00)	0.233 *
**pH 2. Measurement**	7.37 ± 0.12 7.37 (7.10–7.72)	7.31 ± 0.09 7.27 (7.21–7.48)	0.140 *
**PaCO_2_ Measurement 2**	43.06 ± 13.03 40.75 (26.70–72.50)	43.00 ± 14.04 40.90 (22.80–69.70)	0.985 *
**Saturation 02 2nd Measurement**	93.87 ± 10.45 96.00 (60.40–120.00)	89.70 ± 9.54 92.00 (71.00–99.00)	0.167 ^+^
**PaO_2_ Measurement 2**	100.52 ± 41.53 90.75 (48.00–223.00)	81.27 ± 20.29 74.05 (56.90–120.00)	0.140 ^+^
**Lactate Measurement 2**	2.56 ± 2.32 1.40 (0.40–8.50)	3.63 ± 2.44 3.90 (0.60–6.20)	0.316 ^+^
**HCO_3_ 2nd Measurement**	24.15 ± 4.76 25.05 (14.70–29.50)	20.48 ± 5.67 20.85 (14.20–34.00)	0.033 ^+^
**Difference 2. Measurement**	8.02 ± 7.07 5.50 (0.00–25.00)	12.70 ± 6.75 11.90 (3.60–26.00)	0.054 ^+^
**Delta Difference**	−2.76 ± 7.42 −0.50 ((−22.50)–8.50)	6.19 ± 6.15 4.75 ((−1.00)–17.00)	0.001 ^+^

Continuous variables are presented as mean ± standard deviation and median (minimum–maximum). Normality of data distribution was assessed using visual and analytical methods. For group comparisons, variables with normal distribution were analyzed using the Student’s *t*-test, and the corresponding *p* values are indicated with ‘’*’’. Variables with non-normal distribution were analyzed using the Mann–Whitney U test, and the corresponding *p* values are indicated with ‘’^+^’’. All tests were two-tailed, and a *p* value < 0.05 was considered statistically significant.

**Table 7:** ROC Analysis of the Diagnostic Performance of Parameters Related to PaCO_2_–ETCO_2_ Difference in Predicting Mortality.

Test Result Variables	*Cutoff*	AUC	Std. Error	Youden Index J	*p*	Asymptotic 95% Confidence Interval	Asymptotic 95% Confidence Interval	*Sensitivity*	*Specificity*
**Pre-treatment difference**	>6.80	0.383	0.099	−0.27	0.290	0.190	0.577	40.00	33.30
**Post-Treatment Difference**	>8.75	0.713	0.088	0.42	0.054	0.540	0.885	80.00	62.50
**Delta Difference**	>2.90	0.865	0.072	0.71	0.001	0.724	1000	80.00	91.70

AUC: Area Under the Curve AUC). ROC analysis was performed to predict mortality using the PaCO_2_–ETCO_2_ difference at pre-treatment, post-treatment, and delta values. The optimal cutoff point was determined using the Youden index. Sensitivity and specificity values are presented as percentages (%). Statistical significance was accepted at *p* < 0.05.

## Data Availability

The data presented in this study are available on request from the corresponding author due to privacy.
